# Neurexin drives *Caenorhabditis elegans* avoidance behavior independently of its post-synaptic binding partner neuroligin

**DOI:** 10.1093/g3journal/jkae111

**Published:** 2024-05-23

**Authors:** Caroline S Muirhead, Kirthi C Reddy, Sophia Guerra, Michael Rieger, Michael P Hart, Jagan Srinivasan, Sreekanth H Chalasani

**Affiliations:** Department of Biology and Biotechnology, Worcester Polytechnic Institute, Worcester, MA 01605, USA; Molecular Neurobiology Laboratory, The Salk Institute for Biological Studies, La Jolla, CA 92037, USA; Department of Biology and Biotechnology, Worcester Polytechnic Institute, Worcester, MA 01605, USA; Molecular Neurobiology Laboratory, The Salk Institute for Biological Studies, La Jolla, CA 92037, USA; Department of Genetics, University of Pennsylvania, Philadelphia, PA 19104, USA; Department of Biology and Biotechnology, Worcester Polytechnic Institute, Worcester, MA 01605, USA; Molecular Neurobiology Laboratory, The Salk Institute for Biological Studies, La Jolla, CA 92037, USA

**Keywords:** neurexin, neuroligin-independent, behavior, avoidance assays, *C elegans*

## Abstract

Neurexins and their canonical binding partners, neuroligins, are localized to neuronal pre-, and post-synapses, respectively, but less is known about their role in driving behaviors. Here, we use the nematode *C. elegans* to show that neurexin, but not neuroligin, is required for avoiding specific chemorepellents. We find that adults with knockouts of the entire neurexin locus exhibit a strong avoidance deficit in response to glycerol and a weaker defect in response to copper. Notably, the *C. elegans* neurexin (*nrx-1*) locus, like its mammalian homologs, encodes multiple isoforms, α and γ. Using isoform-specific mutations, we find that the γ isoform is selectively required for glycerol avoidance. Next, we used transgenic rescue experiments to show that this isoform functions at least partially in the nervous system. We also confirm that the transgenes are expressed in the neurons and observe protein accumulation in neurites. Furthermore, we tested whether these mutants affect the behavioral responses of juveniles. We find that juveniles (4th larval stages) of mutants knocking out the entire locus or the α-isoforms, but not γ-isoform, are defective in avoiding glycerol. These results suggest that the different neurexin isoforms affect chemosensory avoidance behavior in juveniles and adults, providing a general principle of how isoforms of this conserved gene affect behavior across species.

## Introduction

Animals are exposed to a myriad of chemical cues in their environment. Information about the relevant cues is extracted and processed by nervous systems, leading to behavioral responses. Studies using insects led to the initial descriptions of repellents and attractants; repellents drive movement away from a chemical source, while attractants drive movements toward the source (Dethier 1948; Dethier *et al*. 1960). Despite rapid progress in identifying the relevant chemical cues, we lack a comprehensive understanding of the neuronal pathways that detect chemical cues and drive behavioral changes. Of particular interest are synaptic proteins, where sensory information is processed to modify animal movement. A complete understanding of this process requires a genetically tractable system with a well-defined nervous system that generates robust behavioral readouts.

The nematode *C. elegans* with just 302 neurons connected by identified chemical and electrical synapses (White *et al*. 1986), a complete lineage map (Sulston *et al*. 1983), fully sequenced genome (Sulston *et al*. 1992), and robust behaviors (de Bono and Maricq 2005) is ideally suited to reveal how individual genes affect animal behavior. Studies in this model have identified chemosensory receptors (Troemel *et al*. 1995; Sengupta *et al*. 1996), neuronal signaling molecules (Bargmann 2006), and behavioral models (Pierce-Shimomura *et al*. 1999; Vergassola *et al*. 2007; Iino and Yoshida 2009) required for chemosensory behaviors. Additionally, *C. elegans* has been shown to detect and respond to a wide variety of chemical cues (Ward 1973; Hilliard *et al*. 2002). Notably, this animal has been shown to detect repellents by integrating information from sensory neurons in the head and tail to generate avoidance behaviors (Hilliard *et al*. 2002). We used repulsion behavior to identify a role of a synaptic protein.

Neurexins and neuroligins are transmembrane proteins that are often localized to neuronal synapses and are thought to be critical components of neuronal circuits (Dalva *et al*. 2007). Vertebrates have 3 neurexin genes (*NRXN1-3*) encoding 2 isoforms (α, β), and *NRXN1* uniquely encoding a third γ isoform. Also, these genes have multiple splice sites which could result in thousands of unique proteins from the same locus (Fuccillo *et al*. 2015). While these neurexin isoforms have been shown to affect binding partners and may affect synaptic functions (Fuccillo *et al*. 2015; Südhof 2017; Dai *et al*. 2019), linking specific isoforms with interacting partners or behaviors has been challenging. In contrast, the *C. elegans* genome encodes a single neurexin gene, *nrx-1*, which encodes α and γ isoforms (Haklai-Topper *et al*. 2011; Bastien *et al*. 2023). Moreover, recent studies suggest that this single locus might also generate several isoforms using alternative splicing (Barrett *et al*. 2022). Here we use isoform-specific mutants and show that the γ isoform is selectively required for glycerol avoidance in adults, while the α isoform modifies repellence to the same cue in juveniles.

## Methods

### *C. elegans* strains

All strains were maintained on nematode growth media (NGM) plates with *Escherichia coli*OP50 bacteria as a food source (Brenner 1974). Larval stage 4 (L4) animals were transferred to new plates with bacteria to maintain each strain. All animals were stored at 20°C. A complete list of all strains used in this study and their genotypes are listed in Supplementary Table 1.

### Avoidance assay

We used a single animal avoidance assay as previously described (Hilliard *et al*. 2002). Briefly, young adults (day 1 of adulthood) were transferred to a food-free NGM plate and allowed to crawl around for at least 10 minutes before being tested. A small amount of the aversive test compound dissolved in diH_2_O (∼5 nL) is placed on the tail of the animal while it is moving forward. Animals that generated a reversal with 2 or more body bends or a reversal with an omega turn (large-angled turn) upon exposure to the test compound were scored as an avoidance response. Reversals and omega bends have been previously described (Gray *et al*. 2005). A minimum of 6 plates (with 8–15 animals per plate) were tested over 3 days for each genotype by an investigator who was blinded to the genotype of the animals. An avoidance index was calculated as the ratio of number of avoidance responses to the total number of tests.

### Confocal microscopy

*C. elegans* were anesthetized using 5 µL of 100 mM sodium azide in M9 solution, placed on a 5% agarose pad on glass slides, and covered with a glass coverslip. Transgenic *C. elegans* were analyzed using an inverted Leica TCS SP8 laser-scanning microscope operated by LAS X software. Confocal micrographs were generated by compressing z-stacks as maximum intensity projections in FIJI and figures prepared in Adobe Photoshop CS6 and Adobe Illustrator CS6.

### Statistics

Statistical analysis was performed in R using the car and multcomp libraries. The data were fit to a binomial generalized linear model using the glm function. We chose a binomial model because there are 2 possible behavioral outcomes of exposing a worm to a chemical drop: avoidance or non-avoidance. An ANODEV was subsequently performed on the models using the Anova function. When a significant effect of strain, concentration, or an interaction of both were detected, post hoc analyses were performed using the glht function. If the ANODEV detected no effect, a post hoc analysis was not performed.

An expected value predicted avoidance index (PAI), for each strain and condition, was calculated using the following equation:


$$\log\;\left(\frac{\mathrm{PAI}}{1-\mathrm{PAI}}\right)=X\cdot \beta$$

In this equation, *X* is a design matrix for each strain and concentration. *β* is a vector of fitted coefficients from the binomial generalized linear model. Since we are interested in a worm's response to a stimulus, we can solve the equation for PAI, which will leave us with the following:


$$\mathrm{PAI}=\;\frac{e^{X\cdot \beta }}{1+e^{X\cdot \beta }}$$

PAI is the avoidance index we expect to find on a given worm strain and concentration. Expected values are included on each graph, depicted as a red hash line. The expected value overlaps with average. Average avoidance index is plotted as it easier to compare across genotypes.

## Results

**Fig. 1.**
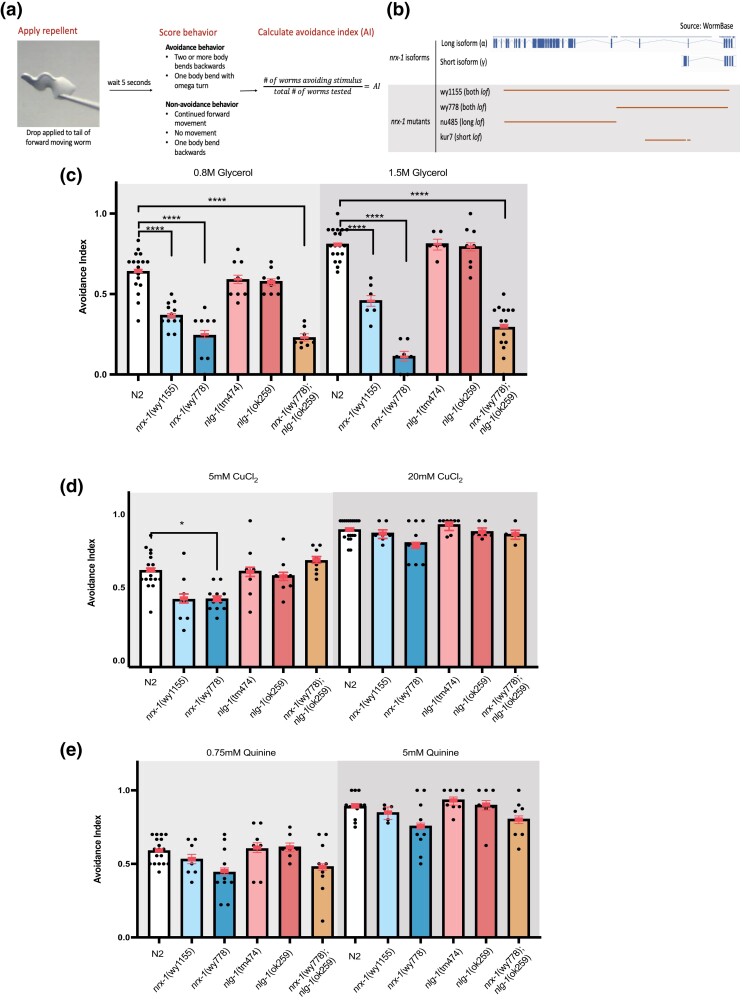
NRX-1 is required for normal glycerol sensation in *C. elegans*. a) Schematic depicts the workflow for an avoidance assay. To perform the avoidance assay, a drop of repellent is applied to a forward moving worm. The worms’ response is scored for avoidance or non-avoidance. Multiple worms are tested per plate to calculate an avoidance index. b) Diagram showing the neurexin mutants used in experiments. The wy1155 allele and wy778 alleles are loss-of-function alleles for both isoforms of neurexin. The nu485 allele removes function of the long isoform while the kur7 allele removes function of the short isoform. c) Plotted values of avoidance index for each strain in response to 1.5 and 0.8 M glycerol. Avoidance index is calculated as number of worms avoiding a drop divided by the number of drops tested per plate. A minimum of 6 plates was tested per condition. Each black dot represents 1 plate of 8–15 worms. Error bars are 95% confidence interval. We found that *nrx-1*(wy1155) and *nrx-1*(wy778) mutants show a decreased avoidance to 0.8 and 1.5 M glycerol. *nlg-1* loss-of-function mutants show no defect. Non-significance was not depicted on the graphs (*P < 0.05, **P < 0.01, ***P < 0.001, ****P < 0.0001). d) *nrx-1*(wy778) mutants showed a decrease in avoidance to 5 mM CuCl_2._ Both *nlg-1* mutants show normal avoidance to 5 and 20 mM copper chloride. e) Both *nrx-1* and *nlg-1* mutants show normal avoidance to 0.75 and 5 mM quinine.

To test the role of neurexin and neuroligin in modifying repellent behaviors, we used a single animal avoidance assay (Hilliard *et al*. 2002). This assay allows us to identify responses of individual animals to test compounds (Fig. 1a, see *Methods*). We tested two independent alleles in both the *neurexin* and *neuroligin* genes. We found that *neurexin* (*nrx-1*), but not *neuroligin* (*nlg-1*) mutants are defective in their responses to the chemorepellent glycerol. Specifically, we found a deletion of the entire *nrx-1* locus (*wy1155*), or the entire C-terminus of both isoforms (*wy778*) was deficient in glycerol avoidance (Fig. 1b). Notably, an allele deleting about half of the *nlg-1* coding sequence (*ok259*) or one deleting exons 7 and 8 of *nlg-1* (*tm474*) did not significantly alter animal repulsion from glycerol. Next, we tested a *nrx-1, nlg-1* double mutant for glycerol avoidance and found that it was not significantly different from the *nrx-1* glycerol avoidance deficit (Fig. 1c). During our statistical analysis, in addition to finding that genotype and chemical concentration influenced glycerol avoidance, we also detected a significant interaction between strain and glycerol concentration, indicating to us that the strains we tested do not uniformly respond to changes in glycerol concentration. Upon further analysis, we determined that this interaction was due to *nrx-1*(wy778) worms’ decreased response to 1.5 M glycerol. To test whether the observed avoidance deficit was specific to glycerol, we analyzed responses to other widely used chemorepellents, copper and quinine. We saw a slight decrease in copper avoidance in *nrx-1*(wy778) worms while the other neurexin mutant, *nrx-1*(wy1155), trended toward lower avoidance too, this difference however did not survive a multiple corrections analysis (Fig. 1d). We found that all *nrx-1* and *nlg-1* alleles along with the double mutants were indistinguishable from wildtype controls in their responses to quinine (Fig. 1e). Taken together, these results show that NRX-1 acts in an NLG-1-independent manner to modify repulsive behavior away from glycerol stimuli, and to a lesser extent, copper. Moreover, this behavioral deficit does not extend to quinine.

**Fig. 2.**
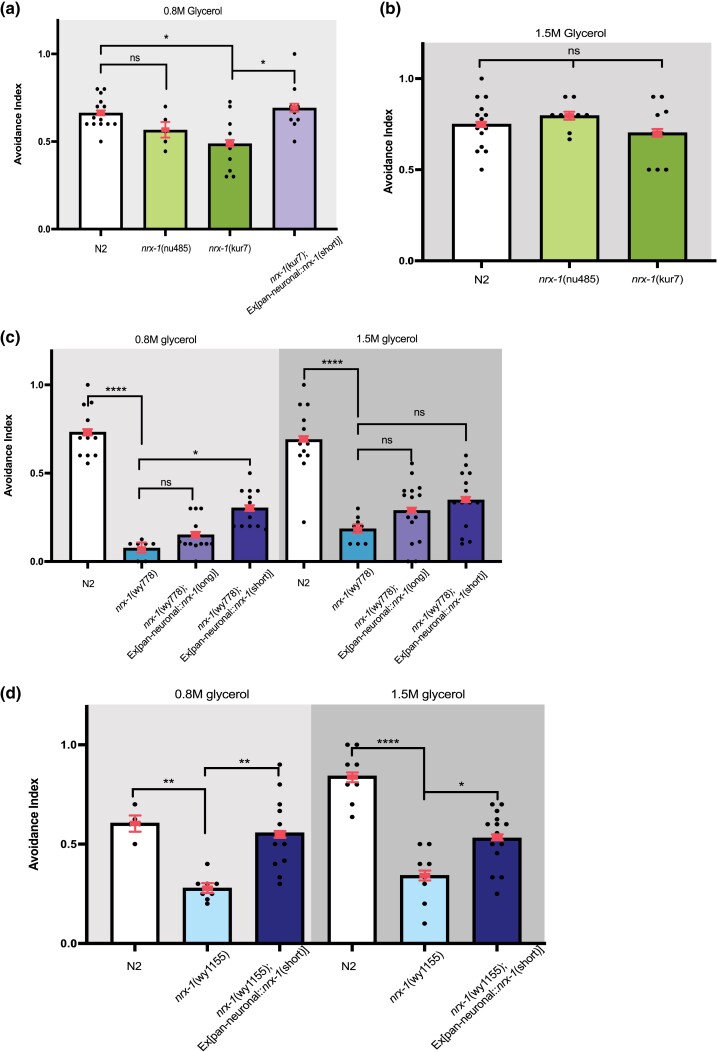
The γ isoform of neurexin primarily modulates glycerol avoidance in adults. a) Plotted values of avoidance index for each strain in response to 0.8 M glycerol, as in Fig. 1. A minimum of 6 plates was tested per condition. Each black dot represents 1 plate of 8–15 worms. Error bars are 95% confidence interval. The nu485 (α-neurexin *lof* only) shows no avoidance defect to glycerol. The kur7 allele (γ-neurexin *lof* only) shows decreased glycerol avoidance at 0.8 M concentration. The defect in the kur7 allele is rescued by expressing β-*nrx-1* under a pan-neuronal promoter (ns = no significance; *P < 0.05, **P < 0.01, ***P < 0.001, ****P < 0.0001). b) *nrx-1*(nu485) and *nrx-1*(kur7) both avoid 1.5 M glycerol at the rate of N2. c) Expressing α-*nrx*-1 in a *nrx-1*(wy778) background showed no rescue effect. Expressing γ-*nrx*-1 in in a *nrx-1*(wy778) background rescued 0.8 M glycerol avoidance. d) Expressing γ-*nrx*-1 in in a *nrx-1*(wy1155) background rescued glycerol avoidance at both glycerol concentrations tested.

The *C. elegans nrx-1* locus encodes multiple isoforms, including orthologs of the α and γ isoforms (Haklai-Topper *et al*. 2011; Barrett *et al*. 2022). To test whether specific isoforms affected glycerol avoidance, we analyzed the responses of isoform-specific mutants *nu485*, which knocked out the long α isoform, and *kur7*, which selectively knocks out the γ isoform. We found that *kur7* mutant animals were defective in glycerol avoidance at a lower glycerol concentration (0.8 M) (Fig. 2a), while *nu485* mutants did not show a significant difference in repulsive behavior at either concentration compared with their wildtype counterparts (Fig. 2, a and b). Next, we tested whether the behavioral deficit in the γ isoform mutants could be rescued by transgenes. We generated transgenic animals expressing the coding sequence for the short γ isoform under a pan-neuronal promoter and analyzed their repulsive responses. We found that neuronal expression of the coding sequence of the γ isoform restored the glycerol avoidance to *kur7* mutants (Fig. 2a). We then tested whether the coding sequence of γ isoform can also restore normal behavior to the *wy778* (allele missing the C-terminus of all isoforms) and *wy1155* (allele knocking out the entire *nrx-1* locus). We found that the expression of the short γ isoform in all neurons was able to partially restore normal behavior to both *wy778* (Fig. 2c) and *wy1155* alleles (Fig. 2d). These results suggest that short γ isoform is selectively required for driving animal avoidance to glycerol. Moreover, this isoform likely functions in the nervous system to affect animal behavior.

**Fig. 3.**
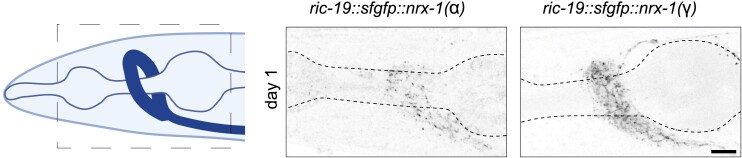
*nrx-1* isoform transgene expression in *C. elegans* neurons. Cartoon of the head of *C. elegans* with pharynx and nerve ring indicated, inset shows location of nerve ring analyzed. Confocal micrograph z-stacks of sfGFP tagged NRX-1(α) or NRX-1(γ) expressed in all neurons (pseudo-colored black/white, scale bar = 20 μm).

Our transgenes included a super folded green fluorescent protein (GFP), which allows for the various isoforms to be directly visualized in animals. We analyzed the expression of the γ isoform and compared it with those observed in animals expressing the long α isoform. We found that both fusion proteins were localized to neurites throughout the nerve ring (transgenes were expressed under pan-neuronal promoters) (Fig. 3). Additionally, we observed protein accumulation in a punctate fashion in the neurites, with some expression in the cell bodies. These data show that our transgenic proteins are indeed expressed within many if not all neurons in the nervous system.

**Fig. 4.**
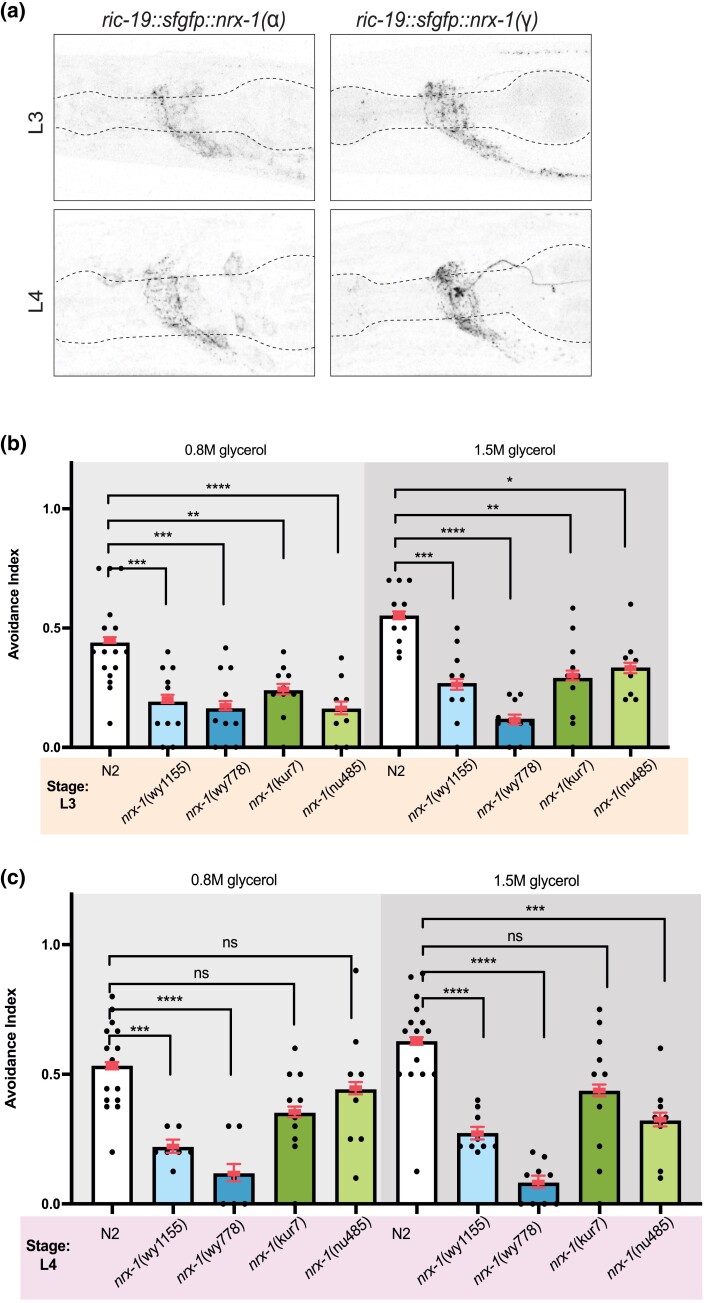
*nrx-1* is required for normal glycerol sensation in L3 and L4 worms. a) Confocal micrograph z-stacks of sfGFP tagged NRX-1(α) or NRX-1 (γ) expressed in all neurons in L3 and L4 animals (pseudo-colored black/white, scale bar = 20 μm). (b) Plotted values of avoidance index for each strain in response to 0.8 and 1.5 M glycerol, as in Fig. 1. A minimum of 6 plates was tested per condition. Each black dot represents 1 plate of 8–15 worms. Error bars are 95% confidence interval. *nrx-1*wy1155, wy778, kur7, and nu485 L3 worms show reduced glycerol avoidance compared to N2 (ns = no significance; *P < 0.05, **P < 0.01, ***P < 0.001, ****P < 0.0001). c) At the L4 stage, wy1155 and wy778 showed reduced glycerol avoidance at both concentrations. The kur7 allele showed no defect while the nu485 allele showed a defect in avoidance at 1.5 M glycerol.

Previous studies indicated that neurexins play an important role in assembling synapses (Dean *et al*. 2003; Dean and Dresbach 2006; De Wit and Ghosh 2016), a critical process in the developing brain. *C. elegans* undergoes a stereotyped developmental program that includes an egg and 4 larval stages (L1–L4) before molting to an adult (Altun and Hall 2009). Moreover, each developmental stage includes specific changes to the nervous system including neurogenesis, migration, pruning, and synaptogenesis (Durbin 1987). To test whether neurexin mutants have phenotypes in developing animals, we first tested whether transgene expression was altered in the developing animal. We observed that expression of both the long α and short γ isoforms were similar to what we observed in the adults (Fig. 4a). Next, we analyzed the avoidance responses of alleles that knockout all isoforms, the long α isoform, or the short γ isoform. We found that in both L3 and L4 animals, *nrx-1* alleles knocking out all isoforms (*wy1155* entire locus knockout, and *wy778* knockout deleting C-terminus of all isoforms) were deficient in glycerol response (Fig. 4, b and c). At the L3 stage, we saw that both the short and long isoforms were individually required for glycerol avoidance (Fig. 4b). But interestingly, at the L4 stage, the long α isoform *lof* (*nu485*), but not short γ isoform *lof* (*kur7*), was defective in glycerol avoidance (Fig. 4, b and c). These results suggest that different neurexin isoforms may mediate glycerol avoidance in a life stage dependent manner.

## Discussion

Our study shows that the short neurexin γ isoform drives glycerol avoidance in adults, while long α neurexin isoform plays a larger role in glycerol avoidance in juvenile worms. Moreover, avoidance deficits in neurexin mutants are the strongest in response to glycerol but there is also a slight defect in copper sensation. Previous studies using cell ablations and calcium imaging have revealed the sensory neurons that are critical to detecting these repellents. While ASH sensory neurons are required for detecting glycerol (Hilliard *et al*. 2005), ASH and ASK neurons are required for quinine (Hilliard *et al*. 2004), and ASH, ADL, and ASE neurons are required for copper avoidance (Sambongi *et al*. 1999). We suggest that the γ isoform might be selectively required in ASH neurons and predict that animals missing this isoform have defective ASH-driven avoidance behaviors, consistent with our observed glycerol avoidance deficit. Also, this hypothesis is consistent with our observation of a lack of avoidance defect for copper and quinine avoidance, since these chemicals can also be detected by additional sensory neurons. However, without cell-specific rescue experiments, it is difficult conclude the site of γ-neurexin activity. It is possible that this isoform might be functional in multiple cells.

Our results also show that NRX-1 functions in an NLG-1-independent manner to modify glycerol avoidance. These data are somewhat surprising since both of these proteins are thought to bind each other (Dean and Dresbach 2006) and function together (Hu *et al*. 2012; Hart 2019). However, additional studies have shown that these proteins can function independently (Maro *et al*. 2015; Kurshan *et al*. 2018; Philbrook *et al*. 2018), or even antagonistically (Hart and Hobert 2018; Lázaro-Peña *et al*. 2018). Our results are consistent with the growing consensus that NRX-1 can have functions that are independent of NLG-1. We speculate that the short γ NRX-1 isoform might interact with other binding partners, including calystenins (*casy-1*), dystroglycans (*dgn-1/2/3*), GluK/GRIK (*glr-1/2/3/5*), GluD1 (*gdh-1*), MDGA1 (*igcm-1*), GABRA1 (*lgc-37*), LRRTM1/2 (*lron-3*, *sma-10*, *hmp-1*, *pan-1*), and others (Hu *et al*. 2012; Maro *et al*. 2015; Hart and Hobert 2018; Kurshan *et al*. 2018; Schaan Profes *et al*. 2024), whose homologs are encoded in the *C. elegans* genome.

We also identify a differential role for the neurexin isoforms during development. Specifically, we find that the long α isoform is required for glycerol avoidance in L4 worms, while the short γ isoform is required for avoiding this cue as an adult. We previously showed that *C. elegans* sensory circuits undergo a juvenile-to-adult transition. Specifically, it was found that while ASH neurons were required to avoid high concentrations of diacetyl in adults, ASH, AWA, and ASK were required to avoid this same cue in juvenile L3 animals (Hale *et al*. 2016). Consistent with these data, we suggest that neural circuit detecting glycerol undergoes a juvenile-to-adult transition and speculate that the long α isoforms are selectively required in the juvenile neural circuit for glycerol repulsion. More broadly, we suggest that the developing adolescent and mature adult brains may utilize different neurexin isoforms to drive behavior.

## Supplementary Material

jkae111_Supplementary_Data

## Data Availability

All behavioral data in this study are included in Supplementary File 1. The code for data analysis can be accessed at https://github.com/shreklab/MuirheadetAl2024_G3. Supplemental material available at G3 online.
