# Supplementary material for: Neurexin drives Caenorhabditis elegans avoidance behavior independently of its post-synaptic binding partner neuroligin
Source: G3 (Bethesda). 2024 May 23;14(8):jkae111. doi: 10.1093/g3journal/jkae111 (PMC11304965; doi:10.1093/g3journal/jkae111)
Supplement: jkae111_Supplementary_Data [file jkae111_supplementary_data.zip › Supplemental_Material_Legends_G3-2024-404974.docx]

**Neurexin drives *C. elegans* avoidance behavior independently of its post-synaptic binding partner Neuroligin**

**Authors**:

Caroline S. Muirhead^1^, Kirthi C. Reddy^2^, Sophia Guerra^1^, Michael Rieger^2^, Michael P. Hart^3^, Jagan Srinivasan^1*^, and Sreekanth H. Chalasani^2*^.

**Affiliations**:

1. Department of Biology and Biotechnology, Worcester Polytechnic Institute, Worcester, MA 01605.

2. Molecular Neurobiology Laboratory, The Salk Institute for Biological Studies, La Jolla, CA 92037.

3. Department of Genetics, University of Pennsylvania, Philadelphia, PA 19104.

* Co-corresponding authors: S.H.C. ([schalasani@salk.edu](mailto:schalasani@salk.edu)) or J.S. ([jsrinivasan@wpi.edu](mailto:jsrinivasan@wpi.edu))

**Short title**:

Neurexin is required for *C. elegans* avoidance.

**Table S1**. Table showing the list of strains used in this study along with their genotypes.

**File S1.** Data from single avoidance assays used in this study.
