# Supplementary material for: Neurexin drives Caenorhabditis elegans avoidance behavior independently of its post-synaptic binding partner neuroligin
Source: G3 (Bethesda). 2024 May 23;14(8):jkae111. doi: 10.1093/g3journal/jkae111 (PMC11304965; doi:10.1093/g3journal/jkae111)
Supplement: jkae111_Supplementary_Data [file jkae111_supplementary_data.zip › Table_S1_G3-2024-404974.pdf]

Table 1: List of strains used this study along with their genotypes.

Strain name	Genotype
TV13570	*unc-119(ed3)* III; *nrx-1(wy778[unc-119(+)])* V
TV22998	*nrx-1(wy1155)* V
VC228	*nlg-1(ok259)* X
IV91	*nlg-1(tm474)* X
IV936	*nrx-1(wy778[unc-119(+)])* V; *nlg-1(ok259)* X
TV22997	*nrx-1(nu485)* V
PTK69	*nrx-1(kur7)* V
IV891	*nrx-1(wy778)* V; ueEx617[ric-19p::nrx-1; unc-122p::RFP]
MPH15	*nrx-1(wy778)* V; hpmEx4[ric-19p::nrx-1(short); myo-2::rfp]
IV1043	*nrx-1(wy1155)* V; hpmEx3[ric-19p::nrx-1(m-short); myo-2::rfp]
IV1066	*nrx-1(kur7)* V; hpmEx4[ric-19p::sfGFP::nrx-1(m- short); myo-2::rfp]
